# Thyroid function tests during nonthyroidal illness syndrome and recovery in acutely ill dogs

**DOI:** 10.1111/jvim.16947

**Published:** 2023-11-30

**Authors:** Timothy A. Bolton, David L. Panciera, Caylie D. Voudren, Matthew I. Crawford‐Jennings

**Affiliations:** ^1^ Department of Small Animal Clinical Sciences, Virginia‐Maryland Regional College of Veterinary Medicine Virginia Tech University Blacksburg Virginia USA

**Keywords:** dog, hypothyroidism, normalization, sick

## Abstract

**Background:**

Nonthyroidal illness syndrome (NTIS) can result in thyroid function test alterations that mimic hypothyroidism. The duration of NTIS‐induced changes in dogs is not well‐described.

**Objectives:**

Document alterations in thyroid function tests during NTIS and recovery, and the time necessary for their resolution.

**Animals:**

From 103 dogs sampled, 25 euthyroid dogs with acute, resolvable illness having a low serum total thyroxine (TT4) concentration on admission were analyzed.

**Methods:**

Prospective observational study. Serum TT4 concentration was measured in 103 dogs within 4 hours of admission. If below the reference interval (RI), subsequent serum samples were obtained every 24 hours from admission until discharge (acute phase) and at 2 weeks and 4 weeks after discharge (recovery phase). Serum samples were submitted for batch measurement of serum TT4, free thyroxine (fT4), total 3,5,3′‐triiodothyronine (TT3), and thyroid‐stimulating hormone (TSH) concentrations.

**Results:**

In the cohort of dogs analyzed, serum TT4, TT3, and fT4 concentrations were below the RI in 100%, 80%, and 16% at admission; 20%, 80%, and 0% at discharge; 4%, 8%, and 0% at 2 weeks; and 0%, 0%, and 0% at 4 weeks, respectively. Serum TSH concentration was within the RI in 100% at admission and discharge, and above the RI in 4% and 12% at 2 weeks and 4 weeks, respectively.

**Conclusions and Clinical Importance:**

Naturally occurring NTIS in dogs induces alterations in thyroid function tests during acute illness and recovery. Measurement of serum TT4 concentration 2 to 4 weeks after discharge or serum fT4 concentration by ED during illness is recommended for accurate assessment of thyroid function in acutely ill dogs.

**Abbreviations**
BCS: body condition score
ED: equilibrium dialysis
fT4: free thyroxine
HPT: hypothalamic‐pituitary‐thyroid
lsM: least squares mean
MCS: muscle condition score
NTIS: nonthyroidal illness syndrome
RI: reference interval
T3: 3,5,3′‐triiodothyronine
T4: thyroxine
TRH: thyrotropin‐releasing hormone
TSH: thyroid stimulating hormone
TT3: total 3,5,3′‐triiodothyronine
TT4: total thyroxine

## 1 INTRODUCTION

Nonthyroidal illness syndrome (NTIS) denotes alterations in the function of the hypothalamic‐pituitary‐thyroid (HPT) axis as a consequence of systemic illness not directly affecting the thyroid gland.^1^, ^2^ Numerous acute and chronic illnesses are responsible for NTIS in dogs, with endocrine, gastrointestinal, renal, neoplastic, and orthopedic diseases commonly reported.^3^, ^4^ After illness resolution, the function of the HPT axis returns to normal. Measurement of serum thyroid hormone concentrations at a single time point in dogs with NTIS demonstrates consistent decreases in total thyroxine (TT4), free thyroxine (fT4), or both, changes that frequently result in an inappropriate diagnosis of hypothyroidism and needless thyroid hormone supplementation in a euthyroid dog.^3^, ^4^, ^5^, ^6^, ^7^ Thyroid function testing should be performed after illness resolution, but because of limited evidence about when such tests normalize in dogs with NTIS, this recommendation is suppositional.

In humans, the relationship between thyroid function tests during NTIS and recovery are well‐described.^1^, ^8^, ^9^, ^10^, ^11^, ^12^, ^13^, ^14^ Conversely, a database search (PubMed and Google Scholar) using keywords “dog,” “nonthyroidal illness,” and “serial thyroid function tests” identified 6 studies (5 acute illness^15^, ^16^, ^17^, ^18^, ^19^ and 1 chronic illness^20^) that have investigated serial thyroid function tests in dogs with NTIS.^15^, ^16^, ^17^, ^18^, ^19^, ^20^ Four studies evaluated dogs having naturally occurring NTIS, but parvoviral enteritis and leishmaniasis were the only disease processes represented and thyroid function testing ceased before illness resolution.^15^, ^16^, ^18^, ^20^ In 2 studies^17^, ^19^ using experimental models of NTIS to evaluate serial thyroid function tests, 1 measured tests through illness resolution.^19^ The relevance of these models to spontaneous illness in dogs is unclear.

Our objective was to document changes in serum thyroid hormones (TT4, fT4, and total 3,5,3′‐triiodothyronine [TT3]) and thyroid stimulating hormone (TSH) concentrations during naturally occurring NTIS and recovery in a cohort of acutely ill, euthyroid dogs with low serum TT4 concentrations. We hypothesized that this cohort of dogs would also have decreased serum TT3, normal serum fT4, and normal serum TSH concentrations, after which any hormone abnormalities would normalize as the illness resolved. Data describing the longitudinal effects of NTIS on the HPT axis will establish recommendations as to when accurate thyroid function testing can be performed.

## 2 MATERIALS AND METHODS

### 2.1 Case selection

The study was approved by the Institutional Animal Care and Use Committee at the Virginia‐Maryland College of Veterinary Medicine (Protocol #19‐168). Client‐owned dogs were eligible for enrollment in this prospective observational study between January 2020 and December 2022. Inclusion criteria consisted of dogs with acute illness (≤1‐week duration) sufficiently severe to require hospitalization. For dogs fulfilling these criteria, a serum TT4 concentration was measured within 4 hours of admission. If serum TT4 concentration was within the reference interval (RI), no more blood samples were obtained and the dog was excluded from the study. If serum TT4 concentration was below the RI, additional blood samples were obtained as outlined below.

Exclusion criteria (no blood sample collected) included cachexia (body condition score [BCS] <5/9 and muscle condition score [MCS] ≤2/4, as defined by the American Animal Hospital Association guidelines and evaluated by a board‐certified internist, TAB),^21^ sighthounds,^22^, ^23^, ^24^, ^25^, ^26^, ^27^ history of chronic disease (previous disease diagnosis requiring ongoing treatment for management of clinical signs or current illness >1‐week duration), and administration of any medication known to affect thyroid function within the 60 days before admission. Excluded medications were glucocorticoids, phenobarbital, potentiated sulfonamides, acetylsalicylic acid, amiodarone, zonisamide, tricyclic antidepressants, toceranib, trilostane, and thyroid hormone replacement.^28^, ^29^, ^30^, ^31^, ^32^, ^33^, ^34^, ^35^, ^36^

### 2.2 Experimental protocol

Each dog having a low serum TT4 concentration within 4 hours of admission received a physical examination by a board‐certified internist (TAB). Diagnostic and therapeutic plans were at the discretion of the attending clinician, but included, at minimum, a plasma biochemistry and CBC. Blood samples for the measurement of serum TT4, TT3, fT4, and TSH concentrations were obtained during the acute and recovery phases of NTIS. The acute phase spanned from admission until discharge. The recovery phase spanned the 4 weeks immediately after discharge.

#### 2.2.1 Acute phase

Blood samples (5 mL into a siliconized glass vacutainer tube) were obtained by lateral saphenous or jugular venipuncture every 24 hours after admission until discharge. After clotting for 30 minutes, each sample was centrifuged at 3000*g* for 10 minutes. Serum was harvested, aliquoted into polypropylene red top tubes, and stored at −80°C.

#### 2.2.2 Recovery phase

Dogs were monitored by their owners for new clinical signs and re‐evaluated 2 weeks and 4 weeks after discharge by 2 clinicians (TAB, CDV). At each visit, dogs had history and physical examinations performed after which 9 mL of blood was collected by lateral saphenous or jugular venipuncture and separated accordingly: 2 mL into an EDTA vacutainer tube, 2 mL into a lithium heparin vacutainer tube, and 5 mL into a siliconized glass vacutainer tube. The lithium heparin and EDTA tubes were submitted for a plasma biochemistry and CBC, respectively, and the siliconized glass tube blood was processed and serum stored as described for the acute phase. No additional blood samples were obtained and the dog was excluded from the study if any of the following occurred during either study phase: diagnosed with a chronic disease, treated with a medication recognized to affect thyroid function, failure to return for any recovery phase follow‐up, euthanasia or death, or enrollment withdrawal by the owner.

As each dog completed the study (4 weeks), batch submission of serum samples was performed. Remaining serum from admission was submitted for measurement of serum concentrations of fT4, TT3, and TSH, and acute and recovery phase serum samples were submitted for measurement of serum concentrations of TT4, fT4, TT3, and TSH. To guarantee the dogs analyzed were euthyroid, additional serum samples were obtained if at least 1 of the following hormone abnormalities was present at 4 weeks: serum TT4 concentration below the RI, serum fT4 concentration below the RI, or serum TSH concentration above the RI. Additional samples were obtained at 4‐week intervals until the abnormalities resolved or 12 weeks was reached. Dogs reaching 12 weeks were classified as euthyroid (normal serum TT4, fT4, and TSH concentrations), subclinical hypothyroid (normal serum TT4 and fT4 concentrations and high serum TSH concentration), or hypothyroid (low serum TT4 and fT4 concentrations and high serum TSH concentration). The subclinical hypothyroid and hypothyroid dogs were excluded from analysis.

Complete blood counts and plasma biochemistry profiles were performed at the Virginia‐Maryland College of Veterinary Medicine clinical pathology laboratory. Serum TT4 concentrations were measured at the Virginia‐Maryland College of Veterinary Medicine clinical pathology laboratory and serum fT4, TT3, and TSH concentrations were measured at the Michigan State University Veterinary Diagnostic Laboratory.

### 2.3 Assays

Serum TT4 concentration was measured using a chemiluminescent immunoassay (Immulite 2000 XPi, Siemens Healthcare Diagnostics, Erlangen, Germany) having a RI of 12.8 to 36.3 nmol/L and a lower limit of detection of 6.4 nmol/L.^37^ Serum fT4 concentration was measured by equilibrium dialysis (ED) using a radioimmunoassay (Antech Diagnostics, Irvine, California) having a RI of 6 to 42 pmol/L.^38^ Serum TSH concentration was measured using a chemiluminescent immunoassay (Immulite 2000, Siemens Healthcare Diagnostics, Llanberis, Gwynedd, UK) having a RI of 0.00 to 0.58 ng/mL.^38^ Serum TT3 concentration was measured using a charcoal separation radioimmunoassay having a RI of 0.8 to 2.1 nmol/L.^39^

### 2.4 Statistical analysis

A sample size calculation using PASS 16 (NCSS, LLC, Kaysville, Utah) indicated that 25 dogs would be needed to detect a mean serum TT4 concentration recovery time of 21.4 days with a 95% confidence interval of 20.4 to 22.4 days.^19^ This sample size estimation assumed a SD of 2.3 days for the recovery time.

Normal probability plots were inspected to determine if numerical variables followed a normal distribution. Normally distributed variables were summarized as means (±SD) whereas skewed variables were summarized as median (minimum, maximum). The number of days to hormone normalization and hospitalization duration were compared between medical and surgical treatment groups using the Wilcoxon rank sum test. Time to serum TT4 and TT3 concentration normalization for all dogs was compared using the Wilcoxon signed rank test. Hormone results (TT4, TT3, fT4, TSH) and ratios (TT3/TT4, fT4/TT4, TT4/TSH) were analyzed (1 outcome at a time) using linear generalized estimating equations and expressed as median (minimum, maximum) and least squares means (SE). The least squares means difference and 95% confidence interval of the difference were calculated.^40^ The linear model specified major time point (admission, discharge, 2 weeks, 4 weeks), case type (medical, surgical), and interaction between major time point and case type as fixed effects with dog identification as the subject of repetition. Correlation within dog was modeled using compound symmetry matrix specification. Interaction between major time point and case type was examined to compare major time points within case type and to compare case type at each major time point. Because all hormone results excluding 1 (serum TT3 concentration at 2 weeks) were not significantly different when comparing medical and surgical cases at each major time point, results from all cases are presented. Where appropriate, *P*‐values were adjusted for multiple comparisons using Tukey's procedure. Analyses were performed using SAS version 9.4 (Cary, North Carolina). Statistical significance was set at *P* < .05.

For statistical analysis, a serum TT4 concentration reported as <6.4 nmol/L was assigned a value of 6.3 nmol/L. Because the magnitude of decrease below 6.4 nmol/L was unknown, this value was selected because it was the next closest result having the same level of precision as the reported value and consequently would not falsely decrease any serum TT4 concentration calculations. A serum TT3 concentration reported as 0.0 nmol/L was assigned a value of 0.1 nmol/L when calculating the TT3/TT4 ratio. This value was selected because it was the next closest result having the same level of precision as the reported value. When calculating the number of days required for serum TT4 and TT3 concentrations to normalize in the dogs in which these hormones were below the RI at completion of the acute phase, 14 or 28 days were added to the duration of the acute phase if normalization occurred at 2 or 4 weeks, respectively.

## 3 RESULTS

### 3.1 Excluded dogs

Of 103 dogs sampled, 76 (73.8%) were excluded during the study for various reasons, including normal serum TT4 concentration at admission (59/76, 77.6%), chronic disease (5/76, 6.6%), death or euthanasia (4/76, 5.3%), owner withdrawal (4/76, 5.3%), and failure to return for any recovery phase follow‐up (4/76, 5.3%).

Two additional dogs completed the study but ultimately were excluded from the cohort analyzed. One dog had a serum TSH concentration above the RI at 4 weeks that persisted at 8 and 12 weeks and was classified as subclinically hypothyroid. The other dog had low serum TT4 and fT4 concentrations and a high serum TSH concentration at 4 weeks that persisted at 8 weeks and 12 weeks and was classified as hypothyroid.

Chronic diseases resulting in exclusion during the study were chronic kidney disease (2), hemic neoplasia (1), diabetes mellitus (1), and Lyme nephritis (1).

### 3.2 Included dogs

Twenty‐five dogs completed the acute phase and 24 dogs completed the recovery phase. One dog returned at 2 weeks only, but serum thyroid hormone concentrations were within the RI and it was included in the cohort analyzed. Two dogs with serum TSH concentrations above the RI at 4 weeks had normal concentrations at 8 weeks. One dog with a serum TSH concentration above the RI at 4 and 8 weeks had a normal concentration at 12 weeks. All 3 were classified as euthyroid and included in the cohort analyzed.

Eighteen unique dog breeds were represented. Fifteen dogs were female (12 spayed and 3 intact) and 10 dogs were male (7 castrated and 3 intact). Mean ± SD age was 5.5 ± 3.4 years and weight was 21.7 ± 12.3 kg.

#### 3.2.1 Disease diagnoses

Sixteen dogs (64%) were diagnosed with a disease managed medically, including gastroenteritis (6/16, 37.5%), traumatic pneumothorax (2/16, 12.5%), bacterial bronchopneumonia (2/16, 12.5%), hepatopathy (2/16, 12.5%), and 1 each (6.3%) of: shock of unknown etiology, parvoviral enteritis, leptospirosis, and pyothorax (MED group). Nine (36%) dogs required surgery for various diseases, including gastrointestinal obstruction (4/9, 44.4%), pyometra (2/9, 22.2%), and 1 each (11.1%) of: gallbladder mucocele, lung lobe torsion, and bacterial epididymitis (SURG group). Six dogs (24%) were treated with carprofen at doses of 1.7 to 2.2 mg/kg for a median of 5.5 doses (range, 1‐17 doses). Median hospitalization duration was 2 days (range, 1‐6 days) for all dogs, 2 days (range, 1‐6 days) for MED group dogs, and 3 days (range, 2‐6 days) for SURG group dogs. No significant difference in the hospitalization duration was found between MED group and SURG group dogs (*P* = .19).

#### 3.2.2 Physical examination abnormalities

Abnormalities at admission included dehydration (12), abdominal pain (7), tachypnea (5), fever (4), ptyalism (4), decreased or absent lung sounds (4), icterus (3), purulent vaginal discharge (2), hypovolemic shock (2), swollen, painful testicles (1), and pulmonary crackles (1). One dog had a normal physical examination. In the recovery phase, physical examination abnormalities resolved and all dogs were reported to be normal or improving with no new clinical signs developing.

#### 3.2.3 Clinicopathologic abnormalities

Abnormalities at admission included inflammatory leukogram (15), hypoalbuminemia (7), increased liver enzyme activities (5), azotemia (5), hyperbilirubinemia (4), and hypokalemia (2). Four dogs had normal CBC and plasma biochemistry. In the recovery phase, all clinicopathologic abnormalities resolved and no new abnormalities developed. No dog had findings consistent with hypothyroidism.

#### 3.2.4 Total thyroxine (TT4)

**FIGURE 1:**
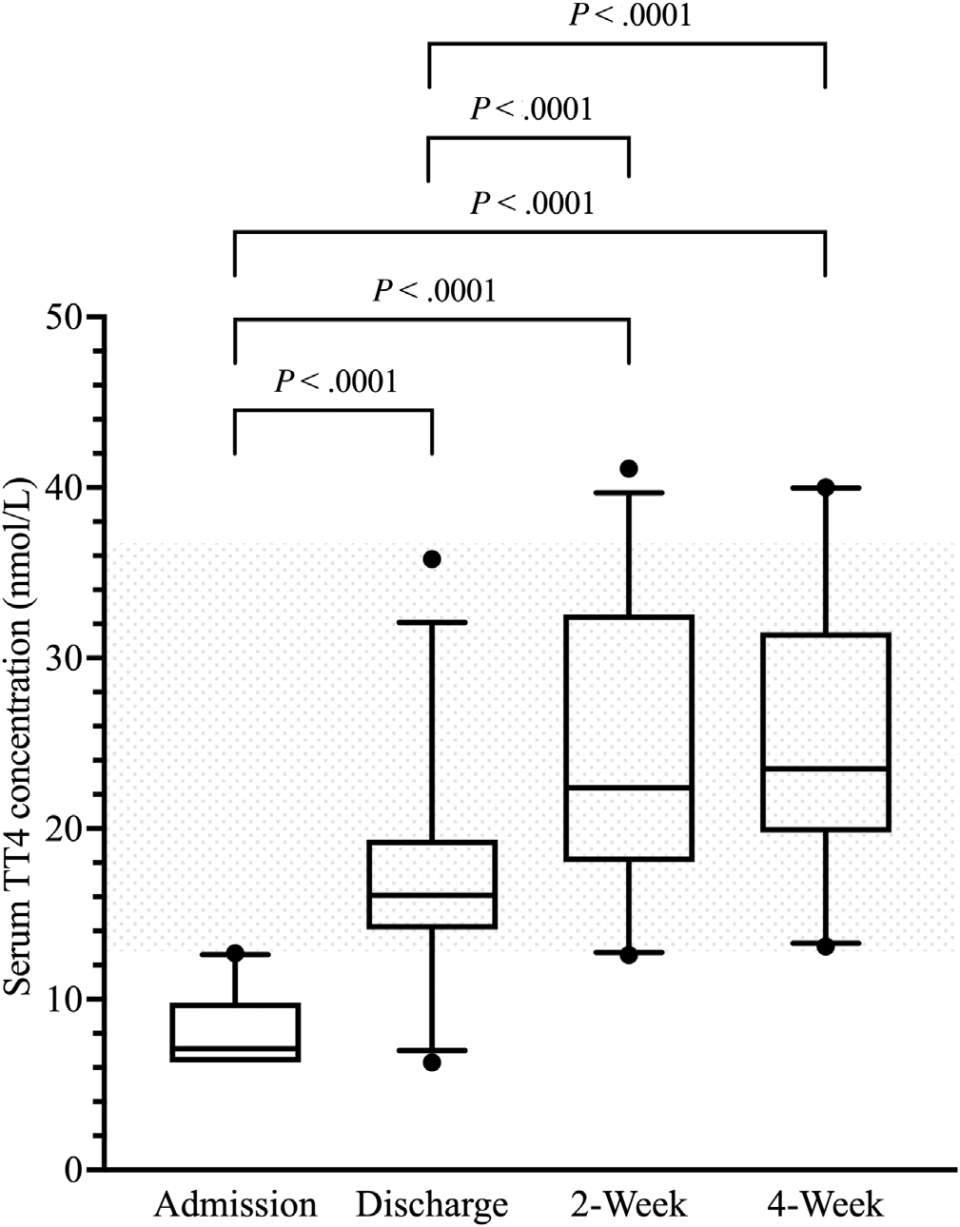
Box‐and‐Whisker plots of the serum total thyroxine (TT4) concentrations (nmol/L) at the 4 major study time points. The shaded area indicates the reference interval.

Median and least squares means (lsM) serum TT4 concentrations at discharge were significantly higher compared with admission. Median and lsM serum TT4 concentrations at 2 and 4 weeks were significantly higher compared with both admission and discharge. Median and lsM serum TT4 concentrations at 2 and 4 weeks were not significantly different (Table 1 and Figure 1). The number of dogs with serum TT4 concentrations of <6.4 nmol/L at admission, discharge, 2 weeks, and 4 weeks were 11, 1, 0, and 0, respectively.

**TABLE 1:** Comparison of serum thyroid hormone concentrations and ratios at the 4 major study time points.

Hormone (RI)	TP 1	Median (range)	LS means (SE)	TP 2	Median (range)	LS means (SE)	LS means ∆	95% CI of ∆	*P*‐value
Total T4	A	7.1 (6.3‐12.7)	7.8 (0.3)	D	16.1 (6.3‐35.8)	16.6 (1.1)	−8.8	−12.0 to −5.6	<.0001
(12.8‐36.3 nmol/L)	A	7.1 (6.3‐12.7)	7.8 (0.3)	2W	22.4 (12.6‐41.1)	24.6 (1.8)	−16.8	−21.6 to −12.0	<.0001
	A	7.1 (6.3‐12.7)	7.8 (0.3)	4W	23.5 (13.1‐40.0)	25.1 (1.7)	−17.3	−21.9 to −12.7	<.0001
	D	16.1 (6.3‐35.8)	16.6 (1.1)	2W	22.4 (12.6‐41.1)	24.6 (1.8)	−8.0	−12.1 to −3.9	<.0001
	D	16.1 (6.3‐35.8)	16.6 (1.1)	4W	23.5 (13.1‐40.0)	25.1 (1.7)	−8.5	−12.6 to −4.5	<.0001
	2W	22.4 (12.6‐41.1)	24.6 (1.8)	4W	23.5 (13.1‐40.0)	25.1 (1.7)	−0.5	−3.5 to 2.5	.98
Total T3	A	0.3 (0.0‐1.2)	0.4 (0.1)	D	0.4 (0.0‐1.4)	0.5 (0.1)	−0.1	−0.2 to 0.0	.20
(0.8‐2.1 nmol/L)	A	0.3 (0.0‐1.2)	0.4 (0.1)	2W	1.1 (0.7‐1.4)	1.1 (0.0)	−0.7	−0.9 to −0.5	<.0001
	A	0.3 (0.0‐1.2)	0.4 (0.1)	4W	1.3 (0.8‐1.8)	1.2 (0.1)	−0.8	−1.0 to −0.6	<.0001
	D	0.4 (0.0‐1.4)	0.5 (0.1)	2W	1.1 (0.7‐1.4)	1.1 (0.0)	−0.6	−0.8 to −0.4	<.0001
	D	0.4 (0.0‐1.4)	0.5 (0.1)	4W	1.3 (0.8‐1.8)	1.2 (0.1)	−0.7	−0.9 to −0.6	<.0001
	2W	1.1 (0.7‐1.4)	1.1 (0.0)	4W	1.3 (0.8‐1.8)	1.2 (0.1)	−0.1	−0.2 to 0.0	.04
Free T4	A	9 (1‐27)	13 (1)	D	19 (9‐38)	21 (1)	−8	−11 to −5	<.0001
(6‐42 pmol/L)	A	9 (1‐27)	13 (1)	2W	19 (9‐37)	21 (2)	−8	−11 to −4	<.0001
	A	9 (1‐27)	13 (1)	4W	15 (7‐35)	19 (1)	−6	−8 to −3	<.0001
	D	19 (9‐38)	21 (1)	2W	19 (9‐37)	21 (2)	0	−3 to 3	1.0
	D	19 (9‐38)	21 (1)	4W	15 (7‐35)	19 (1)	2	0 to 5	.08
	2W	19 (9‐37)	21 (2)	4W	15 (7‐35)	19 (1)	2	0 to 4	.10
TSH	A	0.07 (0.01‐0.32)	0.09 (0.02)	D	0.12 (0.03‐0.58)	0.17 (0.03)	−0.08	−0.14 to −0.03	<.001
(0.00‐0.58 ng/mL)	A	0.07 (0.01‐0.32)	0.09 (0.02)	2W	0.20 (0.05‐0.76)	0.26 (0.04)	−0.17	−0.27 to −0.07	<.001
	A	0.07 (0.01‐0.32)	0.09 (0.02)	4W	0.23 (0.03‐0.92)	0.28 (0.05)	−0.19	−0.31 to −0.08	<.0001
	D	0.12 (0.03‐0.58)	0.17 (0.03)	2W	0.20 (0.05‐0.76)	0.26 (0.04)	−0.09	−0.19 to 0.02	.14
	D	0.12 (0.03‐0.58)	0.17 (0.03)	4W	0.23 (0.03‐0.92)	0.28 (0.05)	−0.11	−0.23 to 0.01	.08
	2W	0.20 (0.05‐0.76)	0.26 (0.04)	4W	0.23 (0.03‐0.92)	0.28 (0.05)	−0.02	−0.09 to 0.04	.77
TT3/TT4 ratio	A	0.04 (0.01‐0.12)	0.05 (0.01)	D	0.03 (0.0‐0.09)	0.03 (0.00)	0.02	0.00 to 0.03	.04
(NRI)	A	0.04 (0.01‐0.12)	0.05 (0.01)	2W	0.05 (0.02‐0.09)	0.05 (0.00)	0.00	−0.02 to 0.02	1.0
	A	0.04 (0.01‐0.12)	0.05 (0.01)	4W	0.05 (0.03‐0.11)	0.05 (0.00)	0.00	−0.02 to 0.02	.99
	D	0.03 (0.0‐0.09)	0.03 (0.00)	2W	0.05 (0.02‐0.09)	0.05 (0.00)	−0.02	−0.03 to 0.00	.01
	D	0.03 (0.0‐0.09)	0.03 (0.00)	4W	0.05 (0.03‐0.11)	0.05 (0.00)	−0.02	−0.03 to −0.01	<.001
	2W	0.05 (0.02‐0.09)	0.05 (0.00)	4W	0.05 (0.03‐0.11)	0.05 (0.00)	0.00	−0.01 to 0.00	.80
fT4/TT4 ratio	A	1.4 (0.2‐3.7)	1.7 (0.2)	D	1.3 (0.6‐2.6)	1.4 (0.1)	0.3	−0.1 to 0.7	.18
(NRI)	A	1.4 (0.2‐3.7)	1.7 (0.2)	2W	0.8 (0.5‐1.6)	0.9 (0.1)	0.8	0.4 to 1.1	<.0001
	A	1.4 (0.2‐3.7)	1.7 (0.2)	4W	0.7 (0.4‐1.4)	0.8 (0.0)	0.9	0.5 to 1.3	<.0001
	D	1.3 (0.6‐2.6)	1.4 (0.1)	2W	0.8 (0.5‐1.6)	0.9 (0.1)	0.5	0.2 to 0.7	<.0001
	D	1.3 (0.6‐2.6)	1.4 (0.1)	4W	0.7 (0.4‐1.4)	0.8 (0.0)	0.6	0.3 to 0.8	<.0001
	2W	0.8 (0.5‐1.6)	0.9 (0.1)	4W	0.7 (0.4‐1.4)	0.8 (0.0)	0.1	0.0 to 0.2	.02
TT4/TSH ratio	A	132.9 (23.1‐1150.0)	183.5 (40.1)	D	166.4 (18.1‐593.3)	164.2 (23.3)	19.3	−53.4 to 91.9	.90
(NRI)	A	132.9 (23.1‐1150.0)	183.5 (40.1)	2W	141.4 (18.3‐525.0)	173.9 (27.5)	9.6	−90.6 to 109.8	.99
	A	132.9 (23.1‐1150.0)	183.5 (40.1)	4W	120.2 (16.2‐1163.0)	217.5 (63.1)	−34.0	−209.2 to 141.1	.96
	D	166.4 (18.1‐593.3)	164.2 (23.3)	2W	141.4 (18.3‐525.0)	173.9 (27.5)	−9.7	−71.3 to 52.0	.98
	D	166.4 (18.1‐593.3)	164.2 (23.3)	4W	120.2 (16.2‐1163.0)	217.5 (63.1)	−53.3	−203.4 to 96.9	.80
	2W	141.4 (18.3‐525.0)	173.9 (27.5)	4W	120.2 (16.2‐1163.0)	217.5 (63.1)	−43.6	−188.0 to 100.7	.87

Abbreviations: ∆, difference; 2W, 2‐weeks; 4W, 4‐weeks; A, admission; CI, confidence interval; D, discharge; fT4, free thyroxine; LS, least squares; NRI, no reference interval; RI, reference interval; T3, 3,5,3′‐triiodothyronine; T4, thyroxine; TP, time point; TSH, thyroid‐stimulating hormone; TT3, total T3; TT4, total T4.

Throughout the study period, the percentage of dogs with serum TT4 concentrations below the RI decreased and the percentage of dogs with serum TT4 concentrations within the RI increased. Fewer than 10% of dogs at any single time point had serum TT4 concentrations above the RI, with all occurring during the recovery phase (Table 2).

**TABLE 2:** Number (%^a^) of cases with serum thyroid hormone concentrations below, within, and above the RI at all study time points.

	Acute phase								Recovery phase	
# of Hospitalized or rechecked dogs	25	25	19	12	6	4	2	25	25	24
Hormone	Admission	Day 1	Day 2	Day 3	Day 4	Day 5	Day 6	Discharge^b^	2‐Week	4‐Week
Total T4 (RI: 12.8‐36.3 nmol/L)										
No. (%) below RI	25 (100%)	16 (64%)	14 (74%)	7 (58%)	3 (50%)	1 (25%)	0 (0%)	5 (20%)	1 (4%)	0 (0%)
No. (%) within RI	0 (0%)	9 (36%)	5 (26%)	5 (42%)	3 (50%)	3 (75%)	2 (100%)	20 (80%)	22 (88%)	22 (92%)
No. (%) above RI	0 (0%)	0 (0%)	0 (0%)	0 (0%)	0 (0%)	0 (0%)	0 (0%)	0 (0%)	2 (8%)	2 (8%)
Total T3 (RI: 0.8‐2.1 nmol/L)										
No. (%) below RI	20 (80%)	21 (84%)	17 (90%)	12 (100%)	6 (100%)	4 (100%)	2 (100%)	20 (80%)	2 (8%)	0 (0%)
No. (%) within RI	5 (20%)	4 (16%)	2 (10%)	0 (0%)	0 (0%)	0 (0%)	0 (0%)	5 (20%)	23 (92%)	24 (100%)
No. (%) above RI	0 (0%)	0 (0%)	0 (0%)	0 (0%)	0 (0%)	0 (0%)	0 (0%)	0 (0%)	0 (0%)	0 (0%)
Free T4 (RI: 6‐42 pmol/L)										
No. (%) below RI	4 (16%)	3 (12%)	0 (0%)	0 (0%)	0 (0%)	0 (0%)	0 (0%)	0 (0%)	0 (0%)	0 (0%)
No. (%) within RI	21 (84%)	22 (88%)	19 (100%)	11 (92%)	6 (100%)	4 (100%)	2 (100%)	25 (100%)	25 (100%)	24 (100%)
No. (%) above RI	0 (0%)	0 (0%)	0 (0%)	1 (8%)	0 (0%)	0 (0%)	0 (0%)	0 (0%)	0 (0%)	0 (0%)
TSH (RI: 0.00‐0.58 ng/mL)										
No. (%) below RI	0 (0%)	0 (0%)	0 (0%)	0 (0%)	0 (0%)	0 (0%)	0 (0%)	0 (0%)	0 (0%)	0 (0%)
No. (%) within RI	25 (100%)	25 (100%)	19 (100%)	12 (100%)	6 (100%)	4 (100%)	2 (100%)	25 (100%)	24 (96%)	21 (88%)
No. (%) above RI	0 (0%)	0 (0%)	0 (0%)	0 (0%)	0 (0%)	0 (0%)	0 (0%)	0 (0%)	1 (4%)	3 (12%)

Abbreviations: fT4, free thyroxine; RI, reference interval; T3, 3,5,3′‐triiodothyronine; T4, thyroxine; TSH, thyroid‐stimulating hormone; TT3, total T3; TT4, total T4.

^a^ % indicates the number of cases below, within, or above the RI divided by the number of cases hospitalized (acute phase) or rechecked (recovery phase) in that day.

^b^ Represents discharge time point from each dog.

During the acute phase, serum TT4 concentrations normalized in 20 dogs, stratified as: 1 day for 9 dogs, 2 days for 2 dogs, 3 days for 6 dogs, 4 days for 1 dog, and 5 days for 2 dogs. Of 5 dogs entering the recovery phase with low serum TT4 concentrations, normalization occurred by 2 weeks in 4 dogs and by 4 weeks in 1 dog. Overall, 24 (96%) and 25 (100%) dogs had normal or increased serum TT4 concentrations by 2 and 4 weeks, respectively. Median time to serum TT4 concentration normalization was 3 days (range, 1‐30 days) for all dogs, 1 day (range, 1‐19 days) for MED group dogs, and 3 days (range, 2‐30 days) for SURG group dogs. No significant difference in the time to serum TT4 concentration normalization was identified between MED group and SURG group dogs (*P* = .06).

#### 3.2.5 Total triiodothyronine (TT3)

**FIGURE 2:**
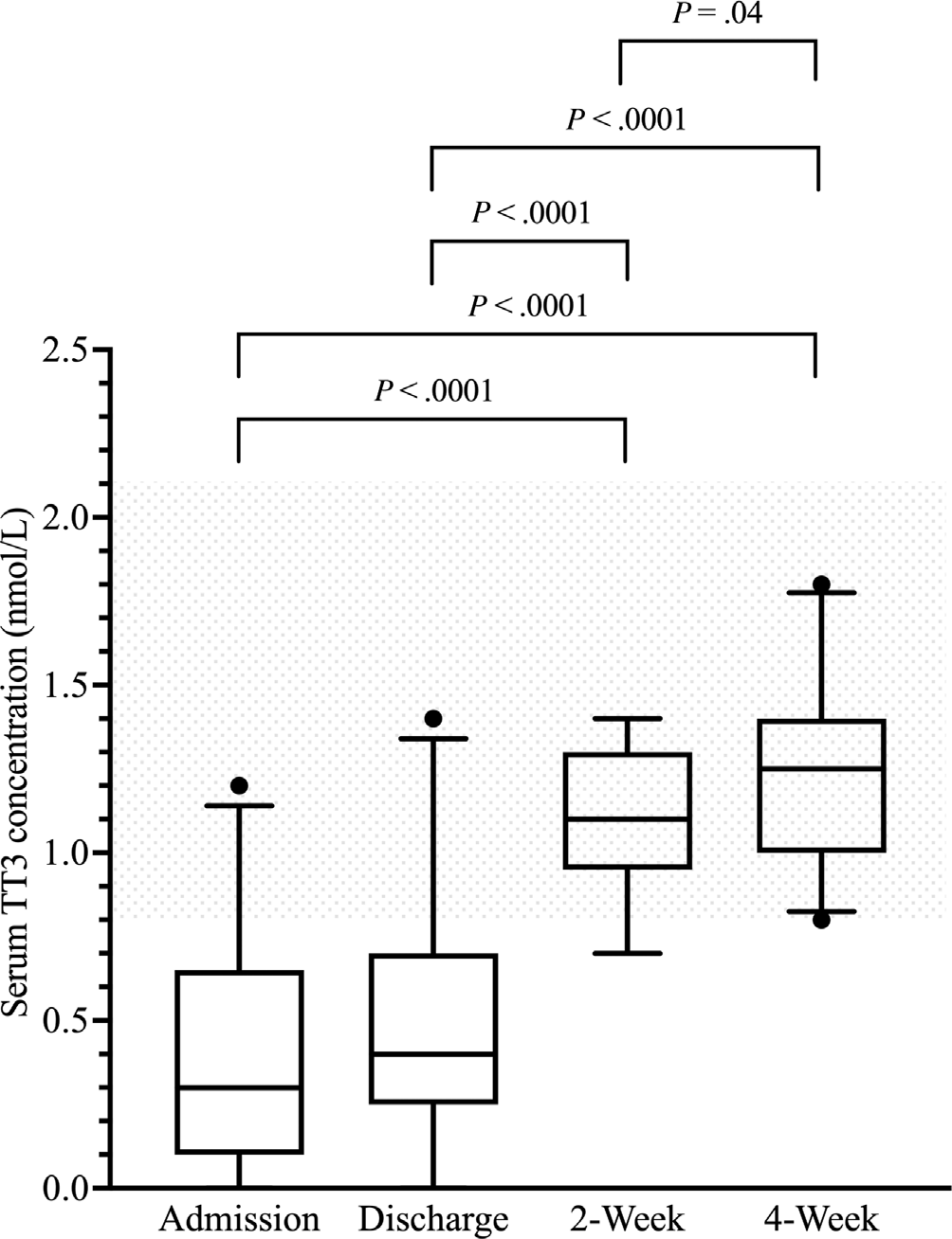
Box‐and‐Whisker plots of the serum total triiodothyronine (TT3) concentrations (nmol/L) at the 4 major study time points. The shaded area indicates the reference interval.

Median and lsM serum TT3 concentrations at 2 and 4 weeks were significantly higher compared with both admission and discharge. Median and lsM serum TT3 concentrations at 4 weeks were significantly higher compared with 2 weeks. Median and lsM serum TT3 concentrations at admission and discharge were not significantly different (Table 1 and Figure 2). The number of dogs with serum TT3 concentrations of 0.0 nmol/L at admission, discharge, 2 weeks, and 4 weeks were 4, 2, 0, and 0, respectively.

Throughout the acute phase, the percentage of dogs with serum TT3 concentrations below the RI increased and the percentage of dogs with serum TT3 concentrations within the RI decreased. Throughout the recovery phase, the percentage of dogs with serum TT3 concentrations below the RI decreased and the percentage of dogs with serum TT3 concentrations within the RI increased. In no dog was the serum TT3 concentration above the RI at any time point (Table 2).

Of 20 dogs with low serum TT3 concentrations at admission, normalization occurred in 3 dogs during the acute phase, stratified as: 1 day for 2 dogs and 2 days for 1 dog. Of 17 dogs entering the recovery phase with low serum TT3 concentrations, normalization occurred by 2 weeks in 16 dogs and by 4 weeks in 1 dog. Overall, 19 (95%) and 20 (100%) dogs had normal serum TT3 concentrations by 2 and 4 weeks, respectively. Median time to serum TT3 concentration normalization was 17 days (range, 1‐30 days) for all dogs, 15.5 days (range, 1‐19 days) for MED group dogs, and 17 days (range, 16‐30 days) for SURG group dogs. No significant difference in the time to serum TT3 concentration normalization was identified between MED group and SURG group dogs (*P* = .17). For all dogs, median time to serum TT3 concentration normalization was significantly longer compared with median time to serum TT4 concentration normalization (*P* < .0001).

#### 3.2.6 Free thyroxine (fT4)

**FIGURE 3:**
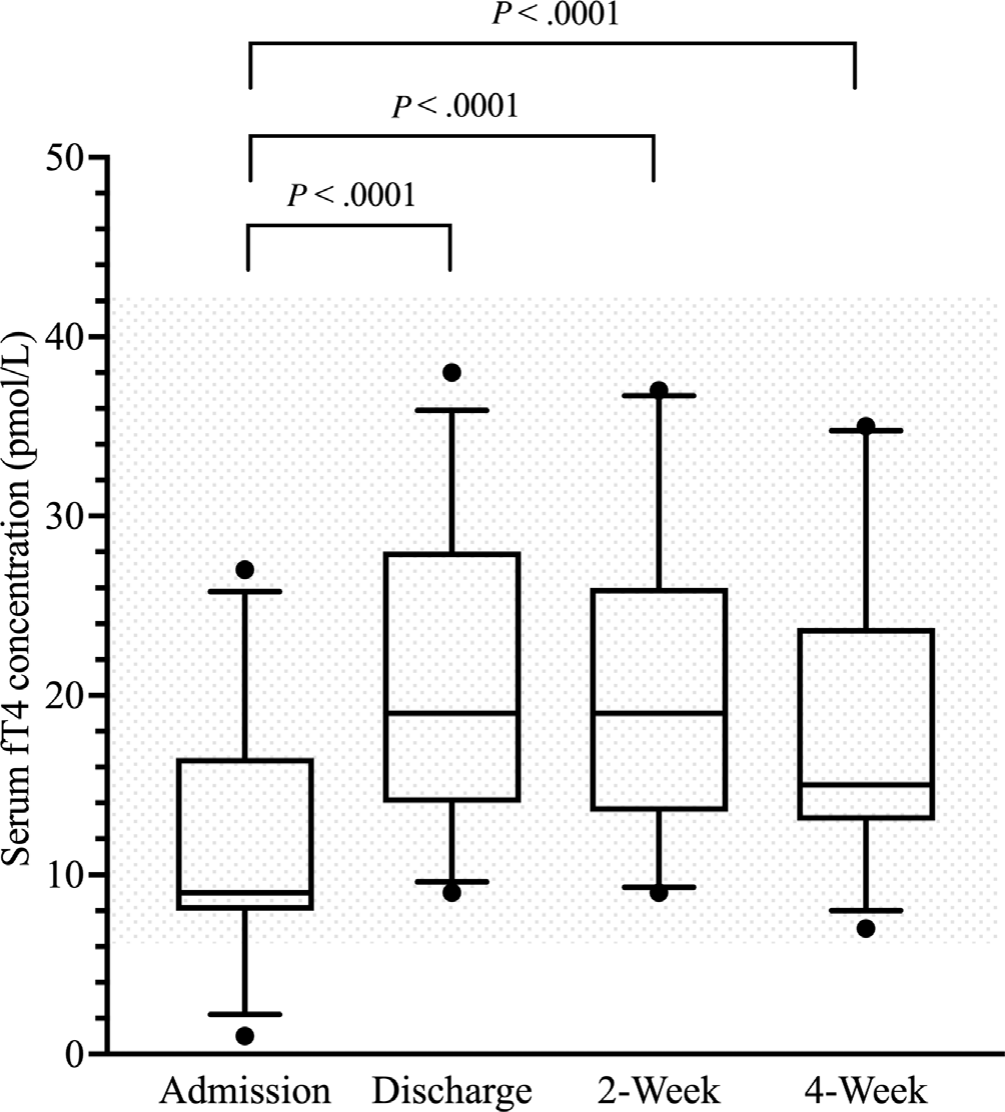
Box‐and‐Whisker plots of the serum free thyroxine (fT4) concentrations (pmol/L) at the 4 major study time points. The shaded area indicates the reference interval.

Median and lsM serum fT4 concentrations at discharge, 2 weeks, and 4 weeks were significantly higher compared with admission. Median and lsM serum fT4 concentrations were not significantly different between discharge and 2 weeks, discharge and 4 weeks, and 2 and 4 weeks (Table 1 and Figure 3).

Throughout the study period, the percentage of dogs with serum fT4 concentrations below the RI decreased and the percentage of dogs with serum fT4 concentrations within the RI increased. One dog had a serum fT4 concentration above the RI, occurring during the acute phase (Table 2).

Of 4 dogs with low serum fT4 concentrations at admission, normalization occurred in all dogs during the acute phase, stratified as: 1 day for 3 dogs and 2 days for 1 dog. Median time to serum fT4 concentration normalization was 1 day (range, 1‐2 days) for all dogs, 1 day (range, 1‐2 days) for MED group dogs, and 1 day (range, 1 day) for SURG group dogs. No significant difference in the time to serum fT4 concentration normalization was identified between MED group and SURG group dogs (*P* = 1.0).

#### 3.2.7 Thyroid‐stimulating hormone (TSH)

**FIGURE 4:**
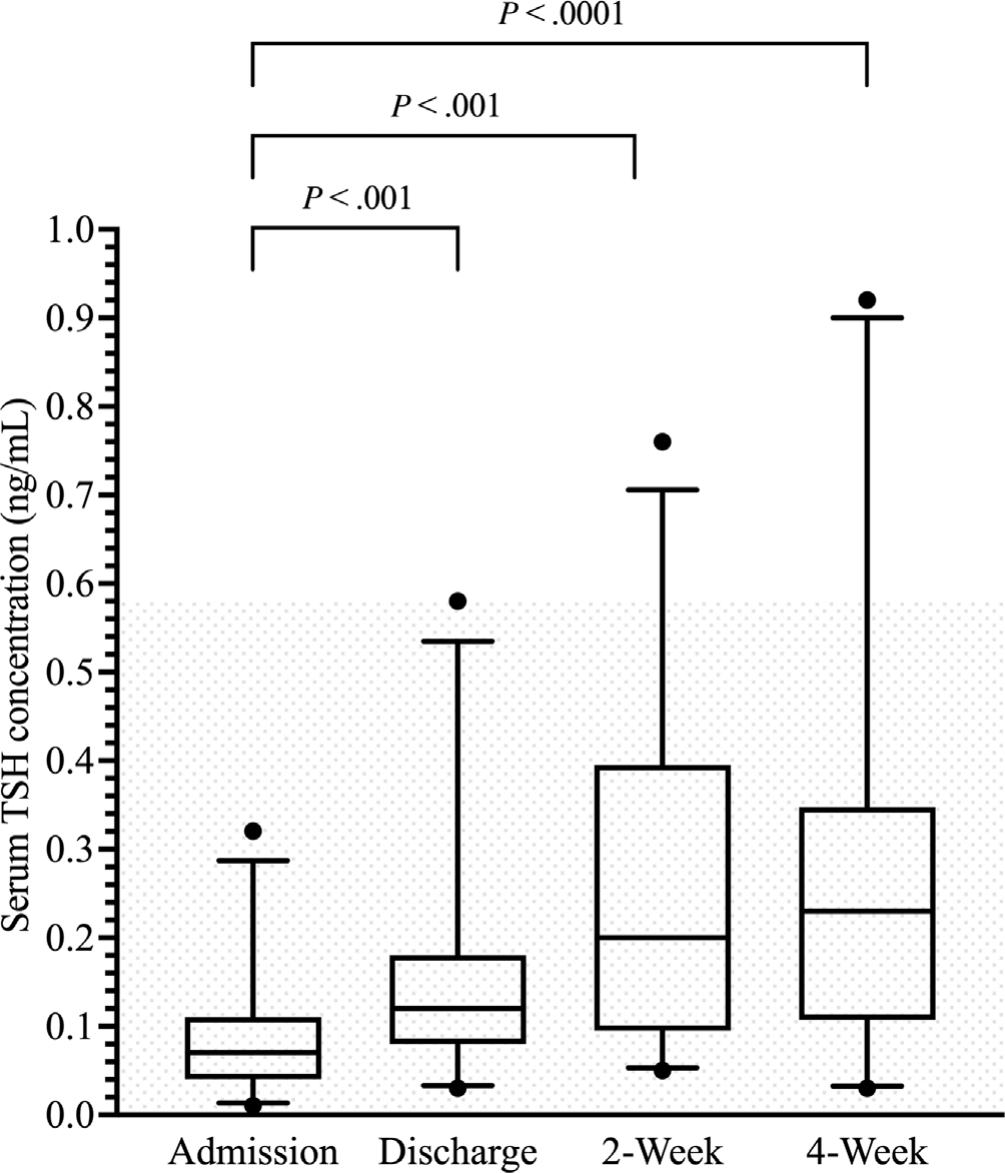
Box‐and‐Whisker plots of the serum thyroid‐stimulating hormone (TSH) concentrations (ng/mL) at the 4 major study time points. The shaded area indicates the reference interval.

Median and lsM serum TSH concentrations at discharge, 2 weeks, and 4 weeks were significantly higher compared with admission. Median and lsM serum TSH concentrations were not significantly different between discharge and 2 weeks, discharge and 4 weeks, and 2 and 4 weeks (Table 1 and Figure 4).

During the acute phase, the percentage of dogs with serum TSH concentrations within the RI was 100%. During the recovery phase, the serum TSH concentration remained largely within the RI but was above the RI in 4% and 12% of dogs at 2 and 4 weeks, respectively (Table 2).

No cases had a low serum TSH concentration at admission, and therefore time to normalization could not be determined.

#### 3.2.8 TT3/TT4 ratio

**FIGURE 5:**
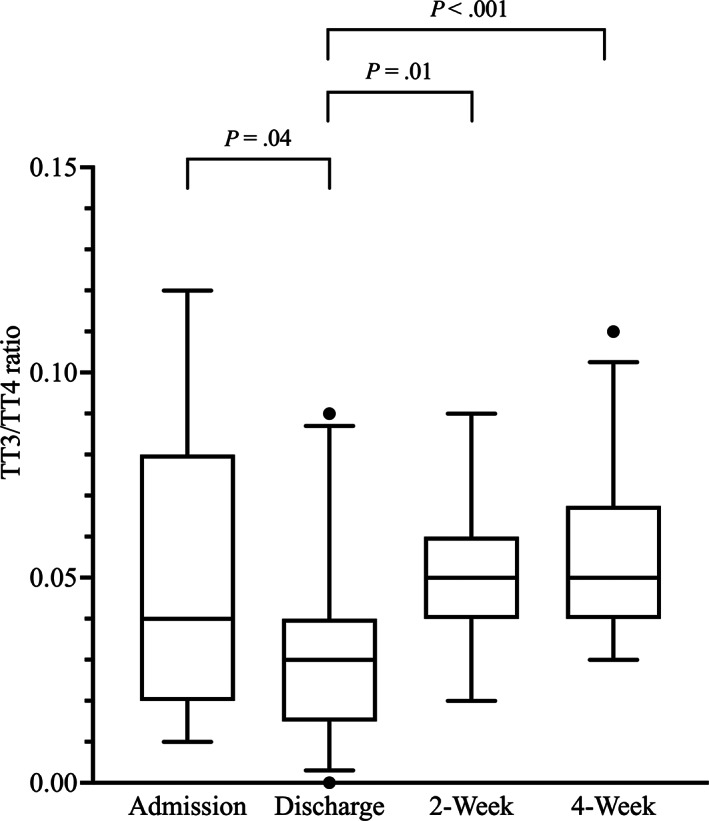
Box‐and‐Whisker plots of the TT3/TT4 ratios at the 4 major study time points.

Median and lsM TT3/TT4 ratios at discharge were significantly lower compared with admission, 2 weeks, and 4 weeks. Median and lsM TT3/TT4 ratios were not significantly different between admission and 2 weeks, admission and 4 weeks, and 2 and 4 weeks (Table 1 and Figure 5).

#### 3.2.9 fT4/TT4 ratio

**FIGURE 6:**
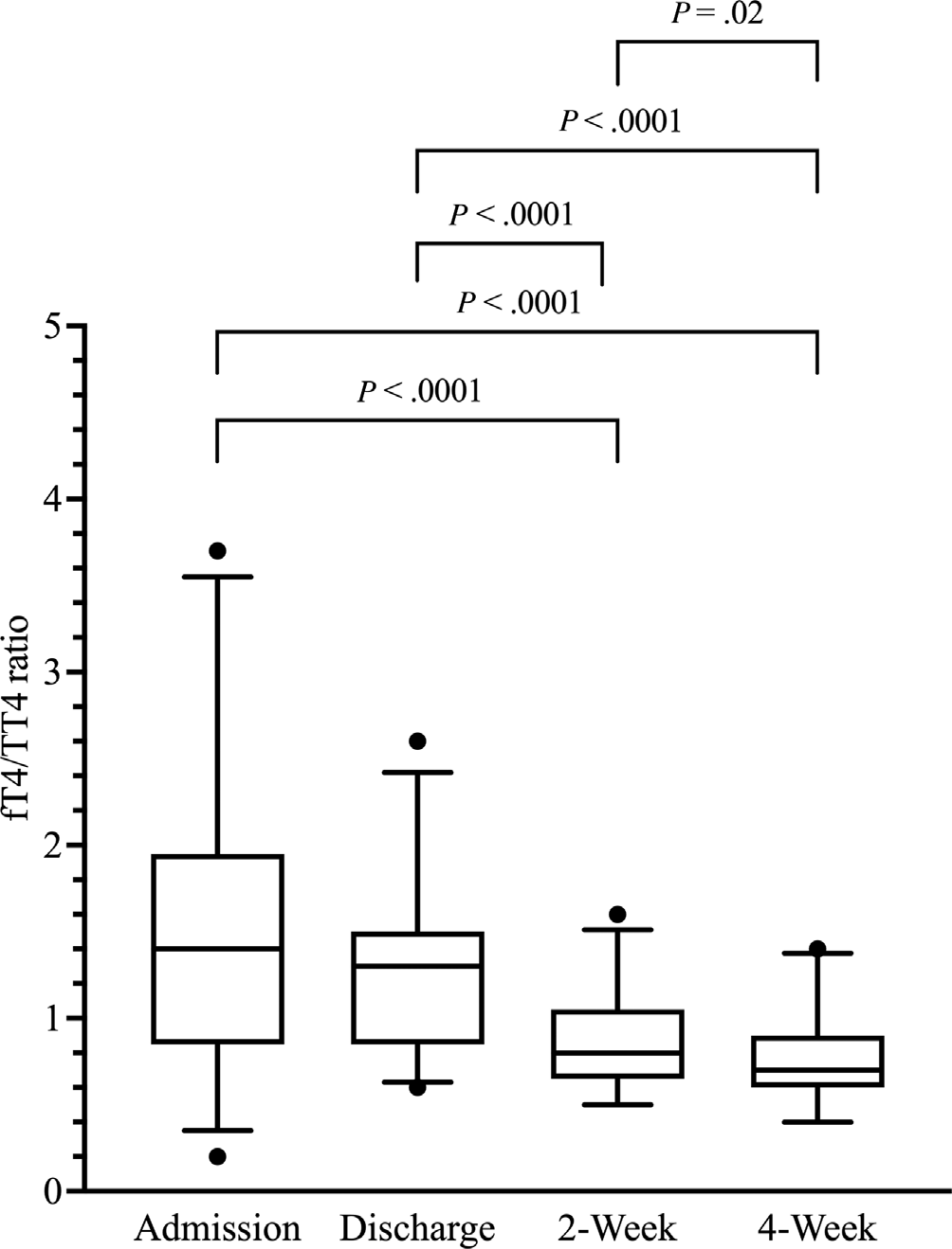
Box‐and‐Whisker plots of the fT4/TT4 ratios at the 4 major study time points.

Median and lsM fT4/TT4 ratios at 2 and 4 weeks were significantly lower compared with both admission and discharge. Median and lsM fT4/TT4 ratios at 4 weeks were significantly lower compared with 2 weeks. Median and lsM fT4/TT4 ratios were not significantly different between admission and discharge (Table 1 and Figure 6).

#### 3.2.10 TT4/TSH ratio

**FIGURE 7:**
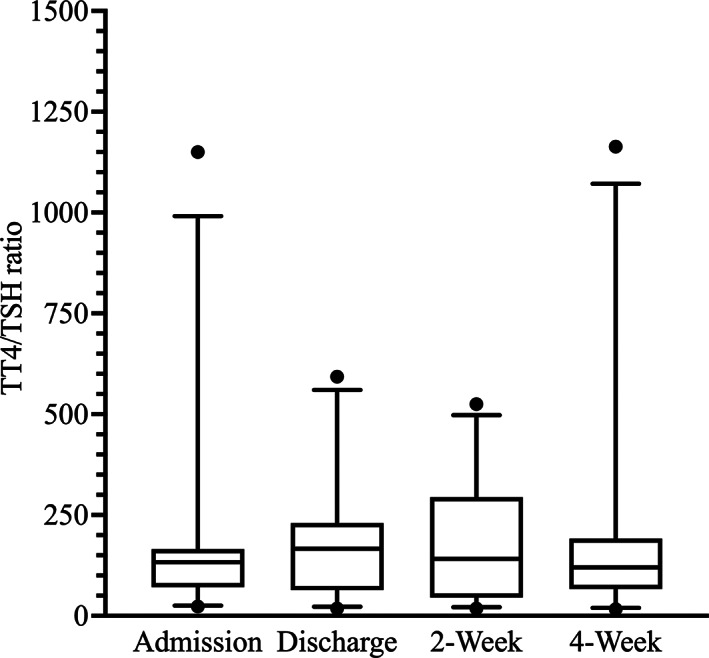
Box‐and‐Whisker plots of the TT4/TSH ratios at the 4 major study time points.

Median and lsM TT4/TSH ratios were not significantly different between any of the time points (Table 1 and Figure 7).

## 4 DISCUSSION

We demonstrated that the low serum TT4 concentration in acutely ill dogs with NTIS recovers rapidly, occurring before discharge in 80% of cases and by 2 to 4 weeks after discharge in all cases. Furthermore, we documented normalization of the serum fT4 concentration within 2 days of admission in all dogs having this hormone below the RI at admission, confirming that serum fT4 concentration by ED is less frequently affected and normalizes even more rapidly than serum TT4 concentration in dogs with NTIS. To prevent a misdiagnosis of hypothyroidism, delaying measurement of the serum TT4 concentration until 2 to 4 weeks after hospitalization for acute illness is recommended. If knowing the functional status of the thyroid gland sooner is essential, serum fT4 concentration should be measured by ED. Lifelong testing and treatment with thyroid hormone supplementation can be avoided by differentiating NTIS from hypothyroidism.

Although our study was not designed to investigate the complex and multifactorial pathophysiologic mechanisms of NTIS, the pattern of hormone changes permits speculation of the mechanisms for the alterations.

Decreases in serum TT4 and TT3 concentrations are common in dogs and humans with NTIS.^3^, ^4^, ^5^, ^6^, ^7^, ^10^, ^12^, ^14^, ^16^, ^20^, ^41^ The decreased serum TT4 and TT3 concentrations at admission in our study could have resulted from suppressed hormone secretion at any level of the HPT axis. In humans, central hypothyroidism, as demonstrated by suppressed TSH secretion in the presence of low circulating thyroid hormones, is a leading contributor to low circulating 3,5,3′‐triiodothyronine (T3) and thyroxine (T4) concentrations and lack of increase in TSH in NTIS.^9^ Loss of the reciprocal relationship between the thyroid hormones and TSH results, in part, from decreased hypothalamic thyrotropin‐releasing hormone (TRH) gene expression, but a direct effect of illness on TSH secretion also contributes.^14^, ^42^, ^43^, ^44^ Decreased secretion of TRH and TSH subsequently decreases T4 and T3 secretion from the thyroid gland. The lower serum TSH concentration at admission compared with other times supports this mechanism as a cause for the low serum TT4 and TT3 concentrations discovered at admission in our study.

From admission to discharge, the serum TT4 concentration increased, serum TT3 concentration remained low and unchanged, and TT3/TT4 ratio decreased, all despite an increase in the serum TSH concentration. Decreased generation of T3 from circulating T4 has been described in models of NTIS as a result of inhibition of type 1 5′‐deiodinase activity or decreased entry of T4 into cells where deiodination occurs.^45^, ^46^, ^47^, ^48^ Decreased 5′‐deiodination of T4 decreases T3 production, prompting a decrease in serum TT3 concentration. Type 1 deiodinase plays a major role in the metabolism of reverse T3 (inactive metabolite of 5‐deiodination of T4), which typically is increased in NTIS, but was not measured in our study. Nevertheless, the lack of increase in serum TT3 concentration at discharge may indicate decreased T3 production via deiodinase inhibition whereas the simultaneous increase in serum TT4 concentration could be secondary to increased thyroidal secretion after resolution of HPT axis suppression. Additional evidence supporting deiodination inhibition might be found between discharge and the recovery phase, where an increase in serum TT3 concentration and TT3/TT4 ratio occurred. These findings, when combined with a lack of increase in the serum TSH concentration between the same time points, could support a reversal of deiodinase inhibition and increased production of T3 from T4 as another cause for the low serum TT3 concentration in NTIS. The effects of NTIS on the serum TT4 and TT3 concentrations appear largely to resolve by discharge and 2 weeks, respectively.

Impaired binding of T4 to plasma transport proteins has been documented as a cause of decreased serum TT4 concentrations in some studies of NTIS.^1^, ^8^, ^9^, ^49^, ^50^ In our study, decreases in the serum TT4 and fT4 concentrations at admission and the increases in both at discharge without a change in the fT4/TT4 ratio over the same time points suggests impaired protein binding does not play a substantial role in the serum TT4 and fT4 concentration alterations in this group of acutely ill dogs with NTIS. Concurrent increases in these hormones are more likely a consequence of increased hormone production secondary to the increased TSH concentration, decreased deiodination secondary to impaired cellular uptake, or both.^41^, ^45^

Humans and dogs with NTIS often have normal or increased serum fT4 concentrations, with low concentrations identified infrequently and most often associated with severe illness.^1^, ^3^, ^4^, ^6^, ^8^, ^9^, ^11^, ^12^, ^16^, ^18^, ^20^ The lower serum fT4 concentration at admission compared with other time points can be the result of a decrease in hormone secretion. This explanation is supported by the increase in serum fT4 concentration at discharge along with a simultaneous increase in the serum TSH concentration. An ED assay was used to measure fT4 in our study. As a reference method for free thyroid hormone measurement, it is unlikely to have underestimated the true serum fT4 concentration.^9^, ^51^

Despite finding a lower serum fT4 concentration at admission relative to all other time points, only 16% of dogs had a result below the RI, a finding consistent with previous studies.^3^, ^4^, ^6^, ^16^, ^20^ Furthermore, normalization of the serum fT4 concentration in these dogs occurred by the time of hospital discharge in all cases. Not only does our study corroborate the findings of other studies of dogs that demonstrated the serum fT4 concentration to be minimally affected in dogs with NTIS, but it also identifies the finding of a rapid return to normal even during acute illness. A serum fT4 concentration measured by ED in a dog with NTIS is likely to be normal and indicate euthyroidism.

During recovery, increases in the serum TT4, TT3, and TSH concentrations along with a lack of change in the TT4/TSH ratio are consistent with a recovery of thyroid hormones caused by increased TSH secretion. Our findings are similar to those found in humans recovering from severe NTIS, where an increase in the serum TSH concentration, sometimes above the RI, occurs in conjunction with increases in the serum TT4 and TT3 concentrations.^10^, ^13^, ^14^ The simultaneous increase in serum thyroid hormone and TSH concentrations throughout recovery strongly supports a reversal of the central hypothyroidism induced during NTIS.

The only study following dogs to illness resolution, and therefore thyroid hormone abnormality normalization, induced illness experimentally by administering repeated doses of IV endotoxin.^19^ Although in the experimental study baseline thyroid hormone concentrations were obtained before endotoxin administration, our study considered the 4‐week time point to represent baseline thyroid hormone results. Both the experimental study and our study identified decreases in serum TT4 (change from baseline, 52%^19^ vs 70%) and TT3 (change from baseline, 62%^19^ vs 77%) concentrations, but the effect was significantly shorter for TT4 (time to normalization, 22 days^19^ vs 3 days) and longer for TT3 (time to normalization, 4 days^19^ vs 17 days) in dogs with spontaneous illness compared with experimentally induced illness. Additionally, the serum fT4 concentration decreased by 53% in our study but increased 73% in the experimental study and the serum TSH concentration was unchanged in the experimental study but decreased 228% in our study.^19^ Our findings indicate that an experimental model of NTIS may not reliably predict changes in serum thyroid hormone and TSH concentrations induced by acute, spontaneous illness, but the differences could be related to physiologic dissimilarities caused by the specific illnesses in each study. Given the numerous disease processes present and the different normalization times for serum TT4 and TT3 concentrations in our study, time to hormone normalization is likely different depending on the illness. Future studies evaluating a larger group of dogs with a single disease process are needed to substantiate this supposition.

Our study had several limitations. First, dogs undergoing surgery with inhalant anesthetics were included. Two studies in dogs have demonstrated that anesthesia and surgery alter serum TT4, TT3, and fT4 concentrations, but any significant change from baseline occurred within 8 to 12 hours after implementation of these interventions.^52^, ^53^ To minimize the impact of anesthesia and surgery on thyroid function tests, dogs had initial blood sampling performed before these interventions and subsequent blood samples were obtained a minimum of 12 hours afterward. Moreover, when serum thyroid hormone concentrations were compared between the dogs managed medically and those undergoing anesthesia and surgery, no significant differences were found except for the serum TT3 concentration at 2 weeks. Thus, it is unlikely that anesthesia and surgery substantially impacted the hormone concentrations in our study. The reason for, and clinical relevance of, the difference in serum TT3 concentration at a single time point between the medical and surgical dogs is unknown. Second, 6 dogs were prescribed a short course of carprofen after disease diagnosis, as deemed appropriate by the attending clinician. Although conflicting data exists about the impact of carprofen on thyroid function tests in dogs, initial blood sampling was performed preceding the carprofen administration in all dogs and the dosages administered do not alter serum TT4, fT4, and TSH concentrations.^54^ Third, we cannot eliminate the possibility that the diagnosed diseases persisted and became chronic or that some dogs already had a concurrent undiagnosed chronic disease because an identical diagnostic evaluation was not performed in every case. This possibility, however, appears unlikely because dogs were reported to be clinically normal, or improving, during the recovery phase and no new clinical signs developed. Moreover, all physical examination and clinicopathologic abnormalities present at admission resolved, as indicated by CBC and plasma biochemistry results in the recovery phase. Fourth, 3 dogs within the cohort studied had at least 1 thyroid function test abnormality at the 4‐week time point that could be consistent with either NTIS or hypothyroidism. Thyroid function testing beyond the 4‐week time point documented resolution of the abnormalities and therefore confirmed euthyroidism. Results from these additional samples, however, were not included in the analysis because so few dogs required sampling at these times and the purpose of these samples simply was to clarify thyroid status. Fifth, the exclusion of dogs having a normal serum TT4 concentration at admission did not permit investigation of the effects of acute illness on the HPT axis in this population of ill dogs and comparison between dogs with NTIS having a serum TT4 concentration within and below the RI. Nonetheless, our study was designed to investigate NTIS defined by a low serum TT4 concentration in a euthyroid dog. Future studies designed to provide this information in a broader group of NTIS dogs can be conducted. Sixth, disease severity has been found to influence the magnitude of changes in thyroid function tests but was not assessed in our study.^55^ Including disease severity in the analysis might allow a more accurate prediction of the effects of NTIS on the HPT axis and time to resolution of these effects. Finally, the frequency of blood sampling during the recovery phase was limited pragmatically to assure client compliance with re‐evaluations. Therefore, the time to thyroid function test normalization likely was overestimated in dogs in which hormone concentrations were still abnormal at discharge. A more precise picture of the recovery phase of NTIS and more precise time to resolution of thyroid hormone abnormalities in dogs potentially could have been elucidated by more frequent blood sampling.

In conclusion, a decrease in the serum TT4 concentration of the magnitude that can result in a misdiagnosis of hypothyroidism occurs in dogs with NTIS.^3^, ^4^, ^5^, ^6^, ^7^, ^16^, ^20^ A serum TT4 concentration measured 2 to 4 weeks after discharge is expected to be free of the suppressive effects of acute illness. If evaluation of thyroid function is desired at an earlier time, serum fT4 concentration by ED is less likely to be affected by acute illness, particularly when measured during hospitalization as the illness begins to resolve.

## CONFLICT OF INTEREST DECLARATION

Authors declare no conflict of interest.

## OFF‐LABEL ANTIMICROBIAL DECLARATION

Authors declare no off‐label antimicrobial use. Although a small portion of dogs in the study were treated with antimicrobials, the antimicrobial selection was at the discretion of the attending clinician and was not a part of the study.

## INSTITUTIONAL ANIMAL CARE AND USE COMMITTEE (IACUC) OR OTHER APPROVAL DECLARATION

Approved by the Virginia Tech University IACUC and Hospital Board of the Virginia‐Maryland College of Veterinary Medicine (Protocol #19‐168). Participation required informed owner consent.

## HUMAN ETHICS APPROVAL DECLARATION

Authors declare human ethics approval was not needed for this study.

## References

[1] Docter R , Krenning EP , de Jong M , Hennemann G . The sick euthyroid syndrome: changes in thyroid hormone serum parameters and hormone metabolism. Clin Endocrinol (Oxf). 1993;39:499‐518. 8252737 10.1111/j.1365-2265.1993.tb02401.x

[2] De Groot LJ . Dangerous dogmas in medicine: the nonthyroidal illness syndrome. J Clin Endocrinol Metab. 1999;84:151‐164. 9920076 10.1210/jcem.84.1.5364

[3] Kantrowitz LB , Peterson ME , Melián C , Nichols R . Serum total thyroxine, total triiodothyronine, free thyroxine, and thyrotropin concentrations in dogs with nonthyroidal disease. J Am Vet Med Assoc. 2001;219:765‐769. 11561650 10.2460/javma.2001.219.765

[4] Mooney CT , Shiel RE , Dixon RM . Thyroid hormone abnormalities and outcome in dogs with non‐thyroidal illness. J Small Anim Pract. 2008;49:11‐16. 17784933 10.1111/j.1748-5827.2007.00418.x

[5] Ramsey IK , Evans H , Herrtage ME . Thyroid‐stimulating hormone and total thyroxine concentrations in euthyroid, sick euthyroid and hypothyroid dogs. J Small Anim Pract. 1997;38:540‐545. 9444634 10.1111/j.1748-5827.1997.tb03313.x

[6] Peterson ME , Melián C , Nichols R . Measurement of serum total thyroxine, triiodothyronine, free thyroxine, and thyrotropin concentrations for diagnosis of hypothyroidism in dogs. J Am Vet Med Assoc. 1997;211:1396‐1402. 9394888

[7] Elliott DA , King LG , Zerbe CA . Thyroid hormone concentrations in critically ill canine intensive care patients. J Vet Emerg Crit Care. 1995;5:17‐23.

[8] Wiersinga WM , van den Berghe G . Nonthyroidal illness syndrome. In: Braverman LE , ed. Cooper DS: Werner & Ingbar's The Thyroid: A Fundamental and Clinical Text. 10th ed. Philadelphia, PA: Lippincott Williams & Wilkins; 2013:203‐217.

[9] Warner MH , Beckett GJ . Mechanisms behind the non‐thyroidal illness syndrome: an update. J Endocrinol. 2010;205:1‐13. 20016054 10.1677/JOE-09-0412

[10] Bacci V , Schussler GC , Kaplan TB . The relationship between serum triiodothyronine and thyrotropin during systemic illness. J Clin Endocrinol Metab. 1982;54:1229‐1235. 7076798 10.1210/jcem-54-6-1229

[11] Chopra IJ . Simultaneous measurement of free thyroxine and free 3,5,3′‐triiodothyronine in undiluted serum by direct equilibrium dialysis/radioimmunoassay: evidence that free triiodothyronine and free thyroxine are normal in many patients with the low triiodothyronine syndrome. Thyroid. 1998;8:249‐257. 9545112 10.1089/thy.1998.8.249

[12] Surks MI , Hupart KH , Pan C , et al. Normal free thyroxine in critical nonthyroidal illnesses measured by ultrafiltration of undiluted serum and equilibrium dialysis. J Clin Endocrinol Metab. 1988;67:1031‐1039. 3182956 10.1210/jcem-67-5-1031

[13] Hamblin PS , Dyer SA , Mohr VS , et al. Relationship between thyrotropin and thyroxine changes during recovery from severe hypothyroxinemia of critical illness. J Clin Endocrinol Metab. 1986;62:717‐722. 3949952 10.1210/jcem-62-4-717

[14] Wehmann RE , Gregerman RI , Burns WH , Saral R , Santos GW . Suppression of thyrotropin in the low‐thyroxine state of severe nonthyroidal illness. N Engl J Med. 1985;312:546‐552. 3881675 10.1056/NEJM198502283120904

[15] Schoeman JP , Goddard A , Herrtage ME . Serum cortisol and thyroxine concentrations as predictors of death in critically ill puppies with parvoviral diarrhea. J Am Vet Med Assoc. 2007;231:1534‐1539. 18020996 10.2460/javma.231.10.1534

[16] Schoeman JP , Herrtage ME . Serum thyrotropin, thyroxine and free thyroxine concentrations as predictors of mortality in critically ill puppies with parvovirus infection: a model for human paediatric critical illness? Microbes Infect. 2008;10:203‐207. 18248764 10.1016/j.micinf.2007.11.002

[17] van Zyl E , Leisewitz AL , Atkinson BK , et al. Serial changes in the concentration of cortisol and thyroid hormones in Beagle dogs infected with *Babesia rossi* . Ticks Tick Borne Dis. 2023;14:102107. 36535203 10.1016/j.ttbdis.2022.102107

[18] Oikonomidis IL , Theodorou K , Papaioannou E , et al. Serial measurement of thyroid hormones in hospitalised dogs with canine parvoviral enteritis: incidence of non‐thyroidal illness syndrome and its association with outcome and systemic inflammatory response syndrome. Vet J. 2021;274:105715. 34252549 10.1016/j.tvjl.2021.105715

[19] Panciera DL , Ritchey JW , Ward DL . Endotoxin‐induced nonthyroidal illness in dogs. Am J Vet Res. 2003;64:229‐234. 12602594 10.2460/ajvr.2003.64.229

[20] Saridomichelakis MN , Xenoulis PG , Chatzis MK , et al. Thyroid function in 36 dogs with leishmaniosis due to *Leishmania infantum* before and during treatment with allopurinol with or without meglumine antimonate. Vet Parasitol. 2013;197:22‐28. 23685064 10.1016/j.vetpar.2013.04.038

[21] Vail DM , Panciera DL , Ogilvie GK . Thyroid hormone concentrations in dogs with chronic weight loss, with special reference to cancer cachexia. J Vet Intern Med. 1994;8:122‐127. 8046675 10.1111/j.1939-1676.1994.tb03209.x

[22] Shiel RE , Brennan SF , Omodo‐Eluk AJ , Mooney CT . Thyroid hormone concentrations in young, healthy, pretraining greyhounds. Vet Rec. 2007;161:616‐619. 17982140 10.1136/vr.161.18.616

[23] Gaughan KR , Bruyette DS . Thyroid function testing in Greyhounds. Am J Vet Res. 2001;62:1130‐1133. 11453491 10.2460/ajvr.2001.62.1130

[24] Sheerer KN , Couto CG , Marin LM , et al. Haematological and biochemical values in North American Scottish deerhounds. J Small Anim Pract. 2013;54:354‐360. 23718887 10.1111/jsap.12086

[25] Panakova L , Koch H , Kolb S , Mueller RS . Thyroid testing in Sloughis. J Vet Intern Med. 2008;22:1144‐1148. 18681922 10.1111/j.1939-1676.2008.0155.x

[26] van Geffen C , Bavegems V , Duchateau L , De Roover K , Daminet S . Serum thyroid hormone concentrations and thyroglobulin antibodies in trained and non‐trained healthy whippets. Vet J. 2006;172:135‐140. 16772138 10.1016/j.tvjl.2005.03.007

[27] Seavers A , Snow DH , Mason KV , Malik R . Evaluation of the thyroid status of basenji dogs in Australia. Aust Vet J. 2008;86:429‐434. 18959530 10.1111/j.1751-0813.2008.00357.x

[28] Daminet S , Ferguson DC . Influence of drugs on thyroid function in dogs. J Vet Intern med. 2003;17:463‐472. 12892297 10.1111/j.1939-1676.2003.tb02467.x

[29] Panciera DL , Refsal KR , Sennello KA , Ward DL . Effects of deracoxib and aspirin on serum concentrations of thyroxine, 3,5,3′‐triiodothyronine, free thyroxine, and thyroid‐stimulating hormone in healthy dogs. Am J Vet Res. 2006;67:599‐603. 16579752 10.2460/ajvr.67.4.599

[30] Daminet S , Croubels S , Duchateau L , et al. Influence of acetylsalicylic acid and ketoprofen on canine thyroid function tests. Vet J. 2003;166:224‐232. 14550731 10.1016/s1090-0233(02)00303-9

[31] Gulikers KP , Panciera DL . Evaluation of the effects of clomipramine on canine thyroid function tests. J Vet Intern Med. 2003;17:44‐49. 12564726 10.1892/0891-6640(2003)017<0044:eoteoc>2.3.co;2

[32] Boothe DM , Perkins J . Disposition and safety of zonisamide after intravenous and oral single dose and oral multiple dosing in normal hound dogs. J Vet Pharmacol Ther. 2008;31:544‐553. 19000278 10.1111/j.1365-2885.2008.00993.x

[33] Harper A , Blackwood L , Mason S . Investigation of thyroid function in dogs treated with the tyrosine kinase inhibitor toceranib. Vet Comp Oncol. 2020;18:433‐437. 31498949 10.1111/vco.12538

[34] Hume KR , Rizzo VL , Cawley JR , Balkman CE . Effects of toceranib phosphate on the hypothalamic‐pituitary‐thyroid axis in tumor‐bearing dogs. J Vet Intern Med. 2018;32:377‐383. 29193327 10.1111/jvim.14882 PMC5787183

[35] Kenefick SJ , Neiger R . The effect of trilostane treatment on circulating thyroid hormone concentrations in dogs with pituitary‐dependent hyperadrenocorticism. J Small Anim Pract. 2008;49:139‐143. 18086154 10.1111/j.1748-5827.2007.00509.x

[36] Liu PM , Zhang XM , Wu W , Li JM , Tang Y . Comparison of cardiac electrophysiologic effects of experimental hypothyroidism and chronic oral amiodarone administration in dogs. Chin Med J (Engl). 1994;107:375‐379. 7924581

[37] Wolff EDS , Bilbrough G , Moore G , Guptill L , Scott‐Moncrieff JC . Comparison of 2 assays for measuring serum total thyroxine concentration in dogs and cats. J Vet Intern Med. 2020;34:607‐615. 32017235 10.1111/jvim.15703 PMC7096613

[38] Ziglioli V , Panciera DL , Troy GC , Monroe WE , Boes KM , Refsal KR . Effects of levothyroxine administration and withdrawal on the hypothalamic‐pituitary‐thyroid axis in euthyroid dogs. J Vet Intern Med. 2017;31:705‐710. 28432797 10.1111/jvim.14711 PMC5435074

[39] Panciera DL , MacEwen EG , Atkins CE , Bosu WT , Refsal KR , Nachreiner RF . Thyroid function tests in euthyroid dogs treated with L‐thyroxine. Am J Vet Res. 1990;51:22‐26. 2105680

[40] Braitman LE . Confidence intervals assess both clinical significance and statistical significance. Ann Intern Med. 1991;114:515‐517. 1994799 10.7326/0003-4819-114-6-515

[41] Kaptein EM , Kaptein JS , Chang EI , et al. Thyroxine transfer and distribution in critical nonthyroidal illnesses, chronic renal failure, and chronic ethanol abuse. J Clin Endocrinol Metab. 1987;65:606‐616. 3116027 10.1210/jcem-65-4-606

[42] Vierhapper H , Laggner A , Waldhäusl W , Grubeck‐Loebenstein B , Kleinberger G . Impaired secretion of TSH in critically ill patients with ‘low T4‐syndrome’. Acta Endocrinol. 1982;101:542‐549. 10.1530/acta.0.1010542 6818804

[43] van den Berghe G , de Zegher F , Baxter RC , et al. Neuroendocrinology of prolonged critical illness: effects of exogenous thyrotropin‐releasing hormone and its combination with growth hormone secretagogues. J Clin Endocrinol Metab. 1998;83:309‐319. 9467533 10.1210/jcem.83.2.4575

[44] Fliers E , Guldenaar SE , Wiersinga WM , et al. Decreased hypothalamic thyrotropin‐releasing hormone gene expression in patients with nonthyroidal illness. J Clin Endocrinol Metab. 1997;82:4032‐4036. 9398708 10.1210/jcem.82.12.4404

[45] Kaptein EM , Robinson WJ , Grieb DA , Nicoloff JT . Peripheral serum thyroxine, triiodothyronine and reverse triiodothyronine kinetics in the low thyroxine state of acute nonthyroidal illness: a noncompartmental analysis. J Clin Invest. 1982;69:526‐535. 6801090 10.1172/JCI110478 PMC371008

[46] Vos RA , de Jong M , Bernard BF , Docter R , Krenning EP , Hennemann G . Impaired thyroxine and 3,5,3′‐triiodothyronine handling by rat hepatocytes in the presence of serum of patients with nonthyroidal illness. J Clin Endocrinol Metab. 1995;80:2364‐2370. 7629231 10.1210/jcem.80.8.7629231

[47] Peeters RP , Wouters PJ , Kaptein E , Van Toor H , Visser TJ , Van den Berghe G . Reduced activation and increased inactivation of thyroid hormone in tissues of critically ill patients. J Clin Endocrinol Metab. 2003;88:3202‐3211. 12843166 10.1210/jc.2002-022013

[48] Hennemann G , Docter R , Friesema EC , et al. Plasma membrane transport of thyroid hormones and its role in thyroid hormone metabolism and bioavailability. Endocr Rev. 2001;22:451‐476. 11493579 10.1210/edrv.22.4.0435

[49] Chopra IJ , Huang TS , Beredo A , Solomon DH , Chua Teco GN . Serum thyroid hormone binding inhibitor in nonthyroidal illnesses. Metabolism. 1986;35:152‐159. 3080653 10.1016/0026-0495(86)90117-4

[50] Wilcox RB , Nelson JC , Tomei RT . Heterogeneity in affinities of serum proteins for thyroxine among patients with non‐thyroidal illness as indicated by the serum free thyroxine response to serum dilution. Eur J Endocrinol. 1994;131:9‐13. 8038911 10.1530/eje.0.1310009

[51] Bennaim M , Shiel RE , Evans H , Mooney CT . Free thyroxine measurement by analogue immunoassay and equilibrium dialysis in dogs with non‐thyroidal illness. Res Vet Sci. 2022;147:37‐43. 35430462 10.1016/j.rvsc.2022.03.016

[52] Wood MA , Panciera DL , Berry SH , Monroe WE , Refsal KR . Influence of isoflurane general anesthesia or anesthesia and surgery on thyroid function tests in dogs. J Vet Intern Med. 2009;23:7‐15. 19138380 10.1111/j.1939-1676.2008.00216.x

[53] Alipour F , Emami MR , Mohri M . Endocrine and oxidative stress characteristics in different anesthetic methods during pneumoperitoneum in dogs. Comp Clin Pathol. 2018;27:1667‐1673.

[54] Sauvé F , Paradis M , Refsal KR , Moreau M , Beauchamp G , Dupuis J . Effects of oral administration of meloxicam, carprofen, and a nutraceutical on thyroid function in dogs with osteoarthritis. Can Vet J. 2003;44:474‐479. 12839241 PMC340170

[55] Giunti M , Troia R , Battilani M , et al. Retrospective evaluation of circulating thyroid hormones in critically ill dogs with systemic inflammatory response syndrome. J Vet Sci. 2017;18:471‐477. 28057899 10.4142/jvs.2017.18.4.471 PMC5746440

